# Destinations Matter: Social Policy and Migrant Workers in the Times of Covid

**DOI:** 10.1057/s41287-020-00326-4

**Published:** 2020-10-27

**Authors:** Nitya Rao, Nivedita Narain, Shuvajit Chakraborty, Arundhita Bhanjdeo, Ayesha Pattnaik

**Affiliations:** 1 School of International Development, University of East Anglia, Norwich, UK; 2 Professional Assistance for Development Action (PRADAN), New Delhi, India; 3 Graham Centre for Agricultural Innovation (An Alliance Between Charles Sturt University and NSW Department of Primary Industries), Bathurst, Australia

**Keywords:** Stranded migrants, Social protection, Citizenship, India

## Abstract

The national lockdown of India announced on March 24th 2020 in response to the COVID-19 pandemic, left millions of migrant labourers stranded in their destinations. Thrown out of their informal labour arrangements in cities and industrial centres, unable to return to their villages in the absence of transportation, they were stranded for over a month with no income, improper housing and often lack of food. This paper discusses the experiences of men migrating from Chakai block, Jamui district, Bihar, to four Indian states, namely, Kerala, Gujarat, Uttar Pradesh and Maharashtra. We compare their experiences across these four destination states in relation to the social policy response following the national lockdown. Most workers are young men (16–35 years old) and their migration pattern is seasonal and circular. The emerging lessons provide inputs for social policy measures related to migrant workers in India.

## Résumé

Suite au confinement national de l'Inde annoncé le 24 mars 2020 en réponse à la pandémie de COVID-19, des millions de travailleurs migrants se sont retrouvés bloqués à l’endroit où ils s’étaient rendus. Après avoir perdu leur emploi informel dans les villes et les zones industrielles, se trouvant dans l’impossibilité de retourner dans leur village faute de moyens de transport, ils ont été bloqués pendant plus d'un mois sans revenus, sans logement convenable et souvent sans nourriture. Cet article évoque les expériences d'hommes en provenance du bloc Chakai, dans le district de Jamui, dans l’État de Bihar, qui ont migré vers quatre États indiens, à savoir le Kerala, le Gujarat, l'Uttar Pradesh et le Maharashtra. Nous comparons leurs expériences respectives dans ces quatre États où ils se sont rendus, à la lumière de la politique sociale mise en place suite au confinement national. La plupart des travailleurs sont de jeunes hommes (16–35 ans) et leur schéma de migration est saisonnier et circulaire. Les leçons que l’on commence à tirer peuvent contribuer aux mesures de politique sociale visant les travailleurs migrants en Inde.

## Introduction

Almost a fourth of India’s population, over 300 million, are migrants settled for various durations in different parts of the country. Of these, close to 30 million (10 per cent) are seasonal migrant workers, 85 percent of them men (Census of India, 2011a), providing cheap labour to the destination economy and bolstering consumption in their home states through remittances. A majority of these migrant workers belong to the poorer states of eastern India—Bihar, Uttar Pradesh, Jharkhand, Odisha and West Bengal—and move to the better-off western and southern states (*Ibid.*). The decision to move is based on mens’ socially constructed ‘provider’ roles, their household conditions and characteristics, including poverty and social identity, and macro-level structural inequalities such as the poor availability of jobs locally (Kothari, 2003; Rao and Mitra, 2013).

The choice of destination is shaped by considerations around wages and working conditions. Migrant workers are aware of the limited state regulation protecting their rights as well as their weak bargaining position, exacerbated by poverty and lack of competitiveness in the labour market, which relegates them to ‘unskilled’ jobs involving manual work. They therefore develop alternative strategies for social support through home-based affiliations, operating in groups that migrate together to the same destination or work in similar settings. Migration itself is viewed as a means of social protection *outside* the parameters of the state. The sense of independence emanating from low-income migrant workers’ roles as breadwinners has meant that they have not organised as a category of ‘internal migrant workers’ to challenge their exclusion from welfare systems and social protection frameworks (Sabates-Wheeler and Feldman 2011; Rao 2011). To this extent, their voice as a legitimate constituency has been largely absent from the public policy space.

In addition to exposing the absence of migrant workers in existing social protection regimes, the pandemic-related lockdown imposed by the Government of India also revealed the underlying inequities in the construction of ‘social citizenship’ itself (Fraser and Gordon 1992). The federal nature of the Indian polity has meant that social policies and regulations relating to migrant workers vary across Indian states. For instance, two critical social protection measures—the right to food (NFSA, 2013) and right to work (MGNREGA 2005)—are place-based and not accessible to mobile populations. Over the past decade, advocates for the formal inclusion of low-income migrant workers in social and economic life have pushed for the portability of social protection entitlements, across and within borders (UNESCO 2013; MacAuslan 2011). This need for the portability of basic rights to food and livelihood has once again come to the fore (Khandelwal 2020).

This paper has two interlinked objectives—one empirical and the second conceptual. Empirically, we seek to explore the experiences of low-income migrant workers, men belonging to Scheduled Tribes (STs) and Other Backward Castes (OBCs), constitutionally designated groups that are recognized as socially and economically disadvantaged, migrating from a remote and poor rural location in the state of Bihar to four different Indian states—Kerala, Gujarat, Maharashtra and Uttar Pradesh. Following the national lockdown from March 25th 2020, these men were stranded, jobless overnight, and desperately trying to return home, in the absence of food or shelter in their destinations. Alongside the physical trauma of being stranded and confronting hardship, most had exhausted their savings and faced the additional social trauma of returning as ‘dependent’ individuals, rather than bringing home money and gifts as the main ‘providers’. Social protection, or its absence, emerged as a critical factor in shaping the experiences, vulnerabilities and disadvantages of low-income migrant workers across destinations, yet has remained largely unstudied.

At a conceptual level, the near-complete failure of extant social protection measures to address the vulnerabilities of stranded migrant workers, allows us to examine how citizenship is constructed and indeed operationalized in practice (Devereux et al. 2011). Whose voice is heard, and whose entitlements are seen as legitimate? Drawing on Fraser’s (1989) articulation of the three moments in the ‘politics of needs satisfaction’, we explore how depoliticised and invisible migrant voices emerged as a legitimate ‘public’ during the lockdown. While embedded in unequal power relations of caste, ethnicity or region, for the first-time their shared interests as productive ‘migrant workers’ and rights-bearing citizens were brought to the fore and coalesced towards the contestation and satisfaction of needs. While claiming their rights may remain a longer struggle, recent announcements as on ‘One Nation, One Ration’, ensuring food entitlements irrespective of place, reflect a starting point in the recognition of migrant workers as a legitimate constituency within social protection frameworks.

This paper draws primarily on qualitative data—focus group discussions with migrant workers in their home villages; telephone interviews when they were stranded at their destinations; and after their return home. The paper is divided into six sections. Section 2 provides an overview of key concepts underlying our analysis. Section 3 delineates the context and methods. Section 4 presents our data in three stages of the migrant experience: pre-lockdown, during the lockdown and after the migrants returned home. Section 5 analyses how the presence or absence of social protection measures and fair working arrangements shaped migrant experiences, as well as the discourses about their ‘needs’ at each stage. Section 6 draws out the contributions of our findings to the wider debates around social protection for migrant workers.

## Conceptual Starting Points

### Migration Analysis in a Social Protection Framework

Income security, diversifying livelihoods, reducing risk and escaping hazards are key drivers of migration for a large section of the poor (Harriss and Todaro 1970). Internal migration itself is hence seen as a form of social protection, driven also by their experiences of poor and grossly inadequate access to state benefits (MacAuslan 2011), which additionally position the poor as ‘dependent clients’ of the state, rather than ‘legitimate claimants’ (Fraser and Gordon 1992). To avoid being seen as ‘clients’, Deneulin (2006, p. 51) sees migration as an “‘exit’ strategy which does not encourage people to ‘voice’ their concerns regarding the government’s incompetence in fulfilling its social duties”. In the process, while migrant workers might improve the wellbeing of their families, they are further excluded from the system of public provisioning.

The small but growing body of international research that locates migration within concerns of social protection starts with conceptualising the fundamental sources of migrant workers’ vulnerabilities as temporal, spatial, socio-cultural and socio-political (Sabates-Wheeler and Feldman 2019, Kabeer 2002). MacAuslan (2011) differentiates these into migrant-specific, migrant-intensified, and bureaucratically imposed ones. Exclusion from ‘source’-based social provisioning, for instance, the right to food at the destination, is a well-researched example of a ‘migrant-specific’ vulnerability, given that migrant workers are unlikely to have political contacts, voice or even identity at the destination (Dreze and Khera 2013). Migrant-intensified vulnerabilities refer to the ways in which migration can exacerbate pre-existing disadvantages, for instance, poverty. A majority of migrant workers are what Breman (1996) calls ‘footloose labour’, engaged in casual wage work with no paid leave or benefits, often over-represented in hazardous, manual work. They have no access to public safety nets, have few reserves, fragile networks and fear of the police further lowers their confidence to overcome discrimination (MacAuslan 2011). Bureaucratically imposed disadvantages refer to official attitudes and perceptions that enhance customary discrimination, in this case, the very invisibility of migrant workers as rights-bearing citizens.

Such an understanding of the interlocking nature of disadvantages that migrants confront helps highlight the relationship of migration with social protection policies, wellbeing and livelihood security, and notions of respectability as individuals and citizens (Rao 2011; Rao and Hossain 2014). Specifically, the nuances of the migrant experience, including the varying needs and vulnerabilities of migrants at origin, in transit and at destination, which remain largely invisible, can be brought to light.

### Citizenship and the Politics of Need

Taking a broader livelihoods and rights approach, acknowledging the multiple disadvantages that migrants confront, Devereux and Sabates-Wheeler conceptualize social protection as including four levels:

… provision measures, which provide relief from deprivation; preventive measures, which attempt to prevent deprivation; promotive measures, which aim to enhance incomes and capabilities; and transformative measures, which seek to address concerns of social justice and exclusion (2008, p. 11).

While these levels can be seen as hierarchical, moving from immediate needs for food and cash to more promotive measures such as improved credit provisioning, or transformational measures including opportunities for asset development or quality education, they can in combination help break unequal social ties and ultimately enable the realization of full social citizenship. The pandemic-related lockdown however re-emphasized that social protection is more than a set of policies and legal formulations and is activated through a political process of negotiation and struggle (Prasad-Aleyamma 2011). In welfare states, with multiple demands on state resources, needs are contested, and needs satisfaction depends on the relative voice of different interest groups or ‘publics’ (Fraser 1989). The focus is not on needs per se, but rather discourses about needs seeking to establish hegemonic interpretations of what constitute legitimate social needs, about the politics of need interpretation.

…comprise three moments that are analytically distinct but interrelated in practice. The first is the struggle to establish or deny the political status of a given need, the struggle to validate the need as a matter of legitimate political concern or to enclave it as a nonpolitical matter. The second is the struggle over the interpretation of the need, the struggle for the power to define it and, so, to determine what would satisfy it. The third moment is the struggle over the satisfaction of the need, the struggle to secure or withhold provision (Fraser 1989, p. 164).

The politicization of needs involves processes whereby some matters break out of zones of discursive privacy and out of specialized or enclaved publics to become foci of generalized contestation. They are however then open to contestation by multiple ‘publics’, the status quoists and those demanding change, with bureaucrats ultimately deciding which needs are to be addressed through policy mechanisms.

### Legal Rights and Their Portability

Clauses (d) and (e) of Article 19(1) of the Constitution of India (1950) enshrines migration as a right to move freely throughout the territory of India and to reside and settle in any part of the country. Given the federal structure of the Indian state, however, there remains ambiguity about which institutions bear the responsibility for protecting this right. The most significant legislation relating to labour migration in India is the Inter-State Migrant Workmen (Regulation of Employment and Conditions of Service) Act 1979. The onus here is on contractors and employers to register workers and uphold their rights, but the small-scale and dispersed nature of migration makes this virtually impossible to implement. Additionally, the Act does not apply to establishments with less than 5 inter-state migrant workers or workers without a contractor, excluding a large number from these safeguards (Srivastava and Sasikumar 2003).

While India collects data on internal migration, the pandemic exposed its limitations with little information readily available on workers’ location or occupations (Ahamad 2020). The latest data from Census 2011 is almost least ten years old and fails to adequately capture short-term circular migration. Recent estimates noted 18,632 migrant workers in Jamui district, our study area, yet the *reverse* migration on June 15th numbered 33,645, pointing to significant undercounting (personal communication from Aspirational District Fellows, Ministry of Home Affairs, Government of India, on June 25th 2020).

The most disadvantaged migrants, those assetless and with weak social networks (Mosse et al. 2002), are also characterized by poor access to social protection more generally, and lack of portability of rights more specifically. As already mentioned, the right to food, implemented through the public distribution system, is based on residence in a particular state and therefore neglects the rights of migrant workers who move across state borders (Drèze and Khera 2013). MacAuslan (2011) suggests making ration cards mobile within and between states to ensure universalization of access. This idea gained momentum during the lockdown and became a symbol for the portability of rights and social provisioning.

## Context and Methods

This section first provides an overview of Chakai, the *source block*, locating it within the context of the broader Jharkhand-Bihar region. It then provides an overview of social policy in the four *destination* states, Kerala, Maharashtra, Gujarat and Uttar Pradesh. We selected these states for two reasons: they are among the states receiving the highest number of migrants from Chakai block (with the exception of neighbouring West Bengal); and the policy variations across these states resulted in starkly different experiences amongst stranded workers. The final subsection focuses on methods used for data collection and analysis.

### Migration from Chakai Block and the Broader Santhal Parganas Region

**Fig. 1:**
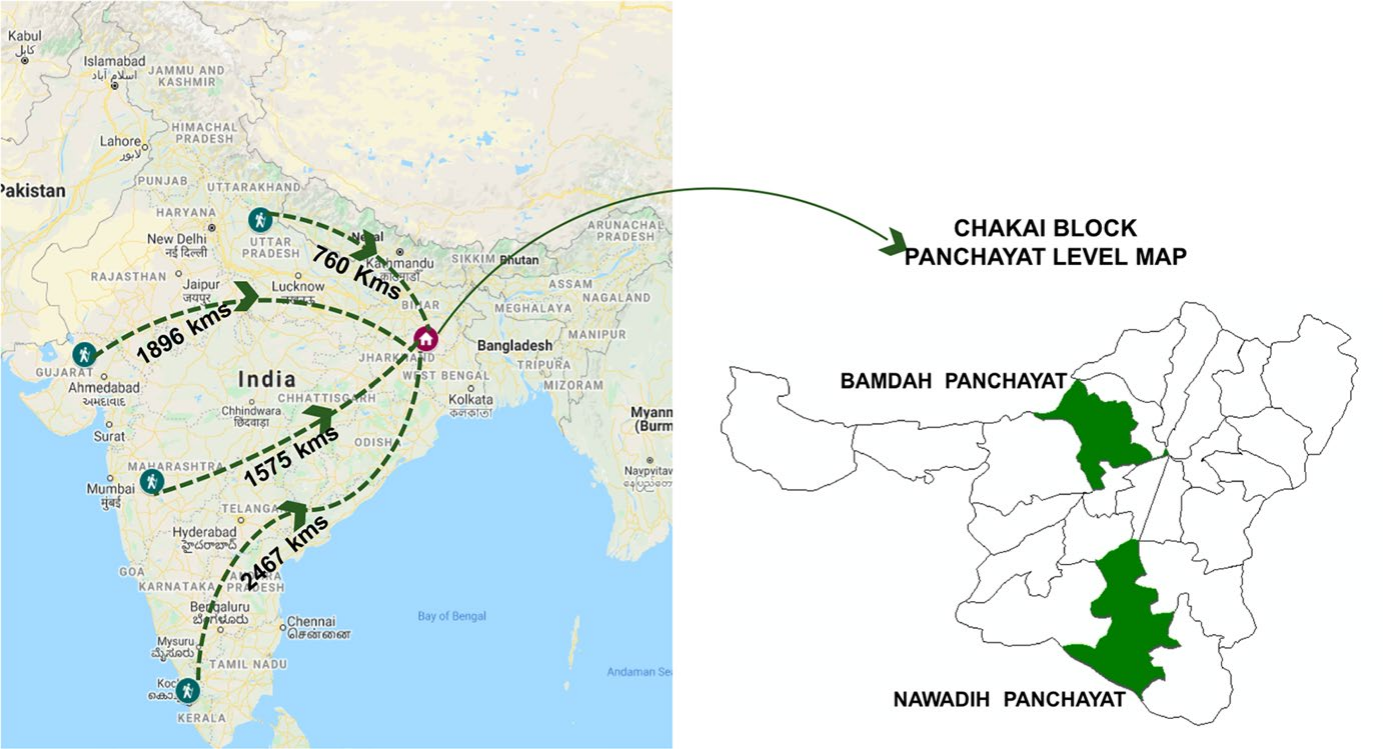
Migration streams from the study location and a map of the study area

Chakai is one of ten blocks in Jamui district, earmarked among the worst-off districts in terms of meeting the SDGs and characterized by left-wing extremist presence. Seventeen percent of the 1.76 million people residing there are tribal, mostly Santhals, in comparison to 4.5% for the district (Census 2011b). The area shares a border with the Santhal Parganas region of Jharkhand and is culturally closely linked with it (Hunter 1907). For subsistence, most families depend on rain-fed agriculture, forest produce collection, agricultural labour and increasingly, seasonal and circular migration. Figure 1 provides a view of our study area.

Santhal migration has a long history, starting with movement of families to the tea gardens of Assam in the late nineteenth century. From the 1980s onwards, with the expansion of irrigation in West Bengal, the demand for agricultural labour, in particular, female labour for paddy transplanting and harvesting, increased (Rao 2008). With rising agrarian distress over the past two decades, the patterns of migration have changed; men now migrating to the relatively better developed states for longer periods of time. The seasonal migration of women has reduced, they manage their family farms and depend on the remittances sent by the men, now seen as the main household breadwinners (Rao and Mitra 2013).

Deshingkar and Akter (2009) suggest that 95 percent of migrants from Bihar were male, migration was highest in the richest and poorest quintiles, and for the poorest, largely driven by distress. In our source villages of Chakai, men aged between 18 and 35 years are away for close to nine months, leaving home after ploughing their paddy lands and returning prior to the next planting season (Rao and Mitra 2013). The lockdown was announced precisely when a few men had returned, but many were left stranded.

### Social Policy in the Four Destination States

State-level variations in policies are crucial to understand labour conditions across India, as migrant workers from the same home state have different experiences based on their destination. The interstate migrant policy index (IMPEX 2019) uses various social, economic and political indicators to calibrate the ease and opportunities for internal migrants to participate in destination states. The scores across states with high migration (Table 1) suggest an urgent need for unified legal frameworks and mechanisms to protect workers.

**Table 1:** The Interstate Migrant Policy Index, 2019

State	Interstate Migration Census (2011a)	IMPEX Score: Overall (out of 100)	IMPEX Score: Social Benefits (out of 100)
Kerala	0.6 million	63	54
Maharashtra	9 million	44	50
Uttar Pradesh	4.06 million	35	0
Gujarat	3.9 million	35	22

*Source* https://indiamigrationnow.org/impex-2019/

While Kerala receives the lowest number of migrants of the four destination states, it is the only Indian state to have introduced provisions to recognize and protect the needs of migrant workers, referring to them as ‘guest workers’ in order to reduce social stigma (Shibu 2020). It has conducted its own migration survey to estimate the numbers of workers leaving from or entering Kerala (Rajan and Zachariah 2020). Its high IMPEX score reflects the introduction of welfare schemes for migrant workers coming into Kerala such as the Migrant Workers Welfare Scheme of 2011 (Aggarwal et al. 2020). However, accessibility remains a challenge, owing to complicated enrolment processes and eligibility criteria of maximum monthly earnings of Rupees 7,500, which is low compared to what migrant workers typically earn (Peter and Narendran 2017). During the lockdown, these specific policies, alongside Kerala’s more general investment in social infrastructure, reduced the hardships faced by migrant workers.

Maharashtra records the highest level of interstate migration, yet the IMPEX scores suggest that its policies pay only moderate attention to migrant workers. The Maharashtra Department of Labour and its Domestic Workers Welfare Board constituted in 2008 have announced various social protection schemes including insurance and educational assistance. However, migrant workers face domiciliary barriers in accessing these schemes, as seen during the lockdown (Kakodkar 2020). Maharashtra’s strong politics of nativism has led to several anti-migrant campaigns (Verma 2011). As the lockdown was partially lifted, the Maharashtra government simultaneously exempted industries from certain central labour laws including the provision under the Factories Act, 1948 limiting working hours (Ram 2020).

In spite of receiving large numbers of migrant workers from across the country, Uttar Pradesh and Gujarat have few measures in place to enforce or protect migrant workers’ rights, also indicated by their low IMPEX scores. During the lockdown, these governments did not announce or provide support to migrant workers. Rather Uttar Pradesh’s cabinet unilaterally passed the Uttar Pradesh Temporary Exemption from Certain Labour Laws Ordinance that exempts establishments from multiple labour laws for a period of 3 years (Ram 2020). The Gujarat government also announced exemptions from provisions under the Factories Act, 1948 relating to weekly hours, daily hours, and intervals for rest (ibid).

### Methods

This study was conducted in three distinct stages: pre-pandemic, during the pandemic-induced lockdown, and after the migrants returned to their homes. The nationwide lockdown brought all travel to a standstill. Some easing of restrictions for migrant workers was announced on May 1st, and later that week the government announced special trains for workers to travel home. Table 2 elaborates these stages, the sample of respondents and the methods adopted.

**Table 2:** Stages of migration, sample and methods adopted

Stage	Methods	Category of respondents	Age group of respondents (years)	Number of respondents
1: Before lockdown: December 2019- January 2020 (n = 250)	Focus group discussions	Migrant Men	18–35	124
		Non-migrating women	18–60	101
	Semi structured interviews	Male migrant workers	18–24 (unmarried); 25–40 (married)	17 (9 unmarried; 8 married)
		Older men (no longer migrant)	50–70	4
		Older women (no longer migrant)	45–60	4
2: During lockdown: March—April 2020 (n = 272)	Phone interviews	Stranded male migrants	18–30	243 (majority unmarried)
	Recordings on *CHIRAGVaani* helpline	Stranded male migrants	18–30	27
3: After returning home: May- June 2020 (n = 85)	Phone interviews	Stranded male migrants	18–32	85 (majority unmarried)

Stage 1: We conducted 17 focus group discussions in four villages across two panchayats to explore the context of migration, the patterns of out-migration and the challenges confronted by the migrant workers and their families. We also conducted semi-structured interviews with 8 older men and women and 17 male migrant workers who had returned to their homes to attend the annual harvest festival, *Sohrai,* to understand changes over the past two decades.

Stage 2: We had launched an Interactive Voice Response System (IVRS) platform, CHIRAGVaani, in the locality, just prior to the lockdown to create a democratic space for the exchange of data and knowledge on sustainable food systems. With the lockdown, migrant workers from Chakai, stranded across the country, started using the number as a helpline. Following a snowballing technique, we sought appointments for telephonic interviews with some of them. Others, seeking help, recorded their experiences on the platform. Of the 411 workers from Chakai stranded across 12 destination states, we identified 272 respondents in the four selected states for this inquiry (Table 3).

**Table 3:** Overview of stranded respondents by destination states

Destination state		Distance from Chakai (kms)	Number of callers (Interviews and recordings)	Number of worker groups represented	Number of workers (approximate)
Kerala		2467	6	4	48
Maharashtra		1575	11	11	53
Uttar Pradesh		760	2	2	26
Gujarat		1896	23	21	145
Total			42	38	272

**Fig. 2:**
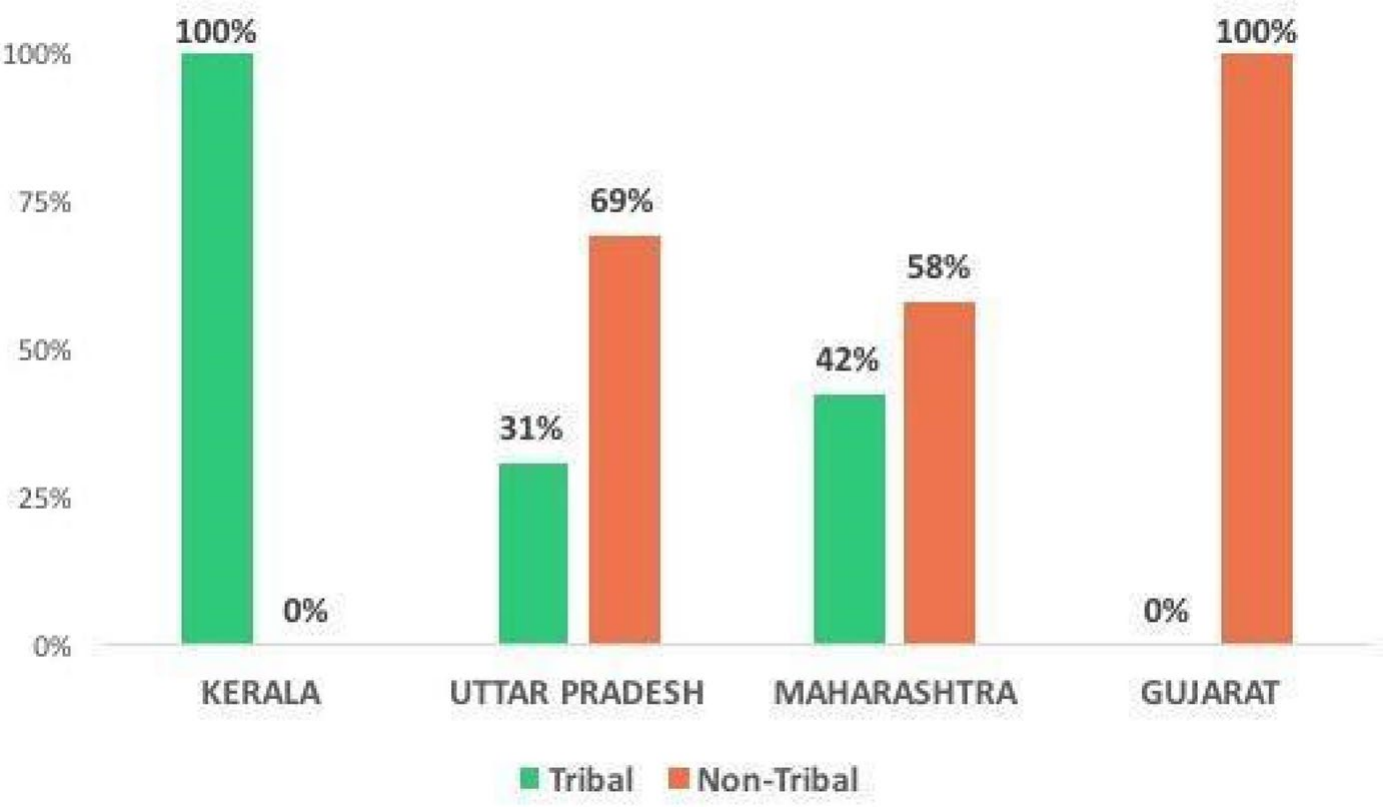
Caste-distribution of the callers across four destinations

Migrant workers stranded in Uttar Pradesh and Maharashtra included both STs and OBCs; whereas those in Kerala were mostly Santhal tribals and those in Gujarat OBCs (Fig. 2).

Stage 3: Towards the end of the lockdown (May–June 2020), many workers began to return home. We conducted follow-up telephonic interviews with 85 workers from the sample interviewed during the lockdown, to ask about their return to Chakai. These workers had also been in touch with us to seek support to travel back home.

All interviews were conducted in Santhali and Hindi and translated into English. They were verified by the research team, and then thematically analysed. A limitation of stage 2 in particular was not just poor phone connectivity, but also the inability to collect specific background data during these interviews, given the distress being faced by the workers. In ideal circumstances, we would have liked to follow-up with the group of respondents we had initially interviewed in the village before the lockdown, but this was not possible.

## The Temporality of Migrant Experiences

This section analyses migrant perspectives and experiences before the pandemic; during the lockdown; and on return home, highlighting their vulnerabilities and perceptions about the destinations, and providing insights into the processes underpinning the ‘politics of needs’.

### Stage 1: Pre-pandemic: Migration Patterns and Vulnerabilities

Men and women between 45 and 70 years reported changes in regional migration patterns over the past two decades. They noted a reduction in both male and female migration to West Bengal as a result of increased farm mechanisation, replacement of labour-intensive varieties of paddy and overall decrease in paddy cultivation due to erratic rainfall. While women go out for daily agricultural wage work around their villages or sell forest produce to earn extra cash, men migrate for unskilled or semi-skilled work in construction sites, factories (bread, biscuits, pipes and rubber making) or mines. Local contractors or agents arrange different kinds of work in distant cities with better wages, not available earlier. A male respondent reported: “As we were not exposed to the labour market, neither were we aware of such work opportunities, nor did we know how to reach that far. Young generations are exploring work as waiters, factory workers, working in big shops etc. which we could not even think about.” This was echoed by the other older men interviewed.

The FGDs with men and women revealed shifts in normative perspectives on migration and work. The younger generation of men, with more education and awareness, appeared reluctant to engage with manual, agricultural work, preferring urban sites. The nature of family responsibilities also shaped the choice of work and destination. Forty-seven percent of the migrant workers interviewed were married, had children and were between 24–40 years of age (Table 2). Two-thirds of them preferred workplaces which offered them some flexibility to travel home. They aspired to earn enough to return to a secure livelihood in the village, where they experienced belongingness and comfort. One said: “If we did the hard work we do in the city here in the village, life would be much more comfortable. So, even though I get respect and young boys ask me about my experiences, I discourage them from going out because there is a lot of suffering. We want to live here, work here and improve our village.”

**Fig. 3:**
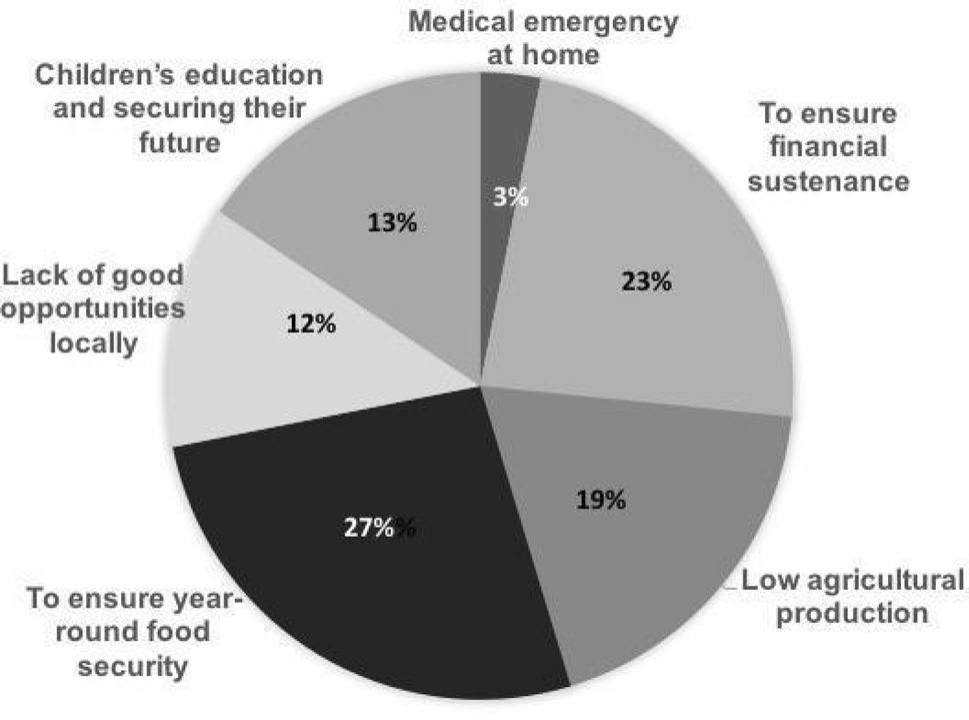
Factors responsible for out-migration

While they preferred to stay near home, they cited lack of local opportunities as a key problem. One of them said, "Whatever work is available in these areas pays very little money and there is already a lot of labour available locally for that kind of laborious work”. Figure 3 outlines the drivers of migration emerging from the interviews with 17 male migrant workers. Of the 64 responses, the two primary drivers were difficulty in ensuring food security (27%) and lack of adequate cash to sustain their families (23%). Low agricultural production, paying for children’s education and securing their future, and attending to medical emergencies at home were other factors driving migration decisions. FGDs with women confirmed that apart from adding to the family’s disposable income, migrants’ remittances strengthened cash flows to deal with exigencies such as roof damages, water seepage, medical emergencies and to meet lumpy investments (otherwise met by borrowing from moneylenders).

**Fig. 4:**
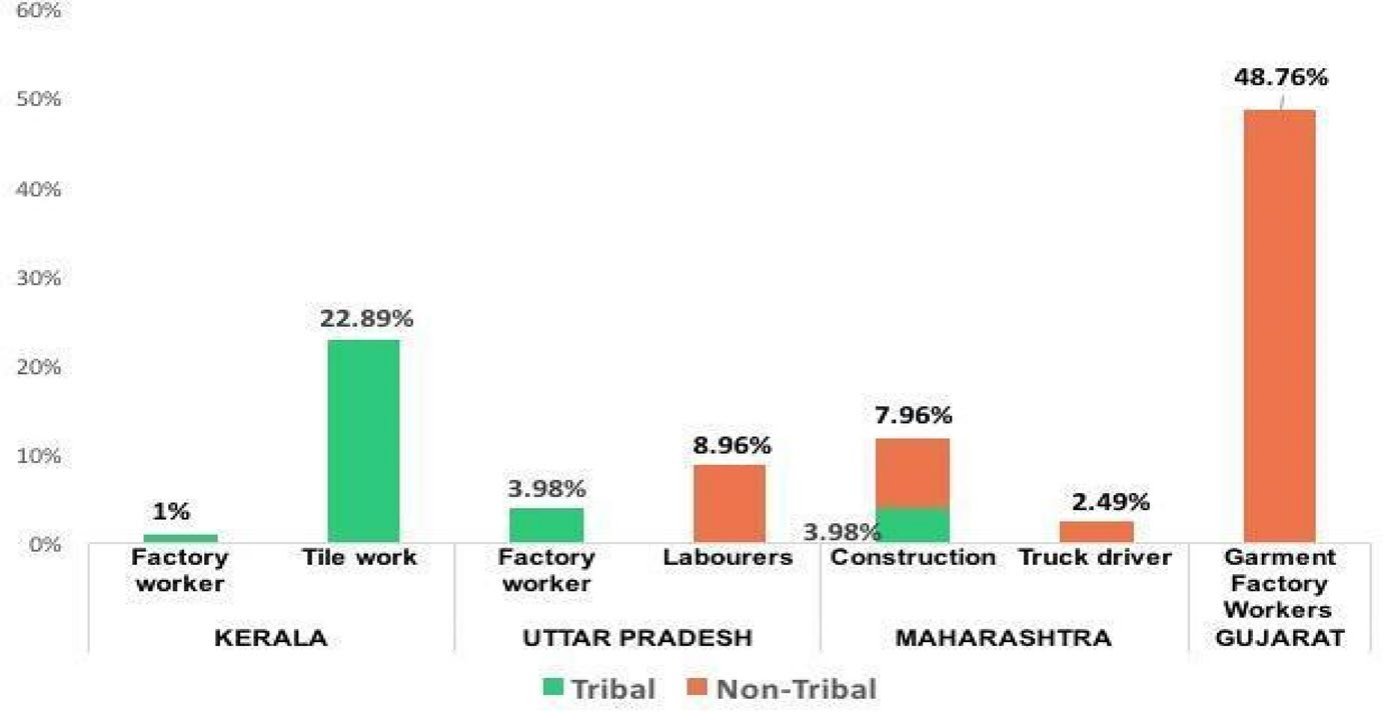
Occupational Distribution of Tribal and Non-Tribal Workers in Destinations (Gathered during Lockdown) (*n* = *195*)

Figure 4 provides the occupational profile of migrants in the selected destination states. Tribal workers with lower levels of education worked mainly as unskilled labourers in factories or construction, while several non-tribal workers engaged in semi-skilled work.

Terms of employment at the destination were negotiated by contractors and employers, with a typical workday being 8–12 h with a lunch break and leave to return home once a year. Food was self-arranged by the workers across destinations, yet there were critical differences across states in terms of working and living conditions (Table 4). While accommodation was shared by groups of 5–10 men, in Kerala and Maharashtra this was provided by the employers. In Gujarat and Uttar Pradesh, the workers had to make their own arrangements, hence often compromised on its quality to save money. This is further linked to the fact that in Gujarat and Uttar Pradesh they were paid daily or piece rates for the work they did, rather than a minimum assured salary per month. If work was not available, they were not paid a wage.

**Table 4:** Terms of Employment Pre-Lockdown

	Gujarat	Uttar Pradesh	Maharashtra	Kerala
Mode of payment	Daily wages	Daily wages	Salaried	Salaried
Workers perception of wages	Low	Low	High	High
Accommodation Arranged by	Workers	Workers	Employers	Employers
Healthcare benefits	Unavailable	Unavailable	Unavailable	Government Health Insurance

Kerala was the only state that had arrangements for the healthcare of migrant workers. In Gujarat and Uttar Pradesh, workers also complained of the lack of support from employers—not receiving payments on time or the use of abusive language in speaking to them. Despite these problems, none of them articulated the need for social protection or better regulation of their destination workplaces. Rather than making claims vis-à-vis the state as a legitimate constituency of migrant workers, they expressed pride in their hard work to improve the wellbeing of their families.

### Stage 2: During the COVID19-Lockdown: Being Stranded

The repeated extension of the lockdown left migrant workers stranded without work, cash and food regardless of their social profile. The nation-wide helplines set up by the Stranded Workers Action Network (SWAN) interacted with about 34,000 workers by 5th June and reported that 50% of the workers had rations left for less than 1 day, and 64 percent had less than 100 rupees left with them (SWAN, 2020). These reports also reflect lack of proper housing, sanitation, electricity, cooking fuel and drinking water facilities. Workers were also worried about their families in the villages: food consumption had reduced with more than half the households eating fewer items and less frequently (PRADAN et al. 2020).

Table 5 provides an overview of the issues reported by workers across the study states and the differential responses of the state governments. Stranded workers in our study destinations reported a variety of migrant-specific disadvantages as they navigated the crisis, especially due to unfamiliarity with cities, language barriers and regional exclusion. Migrant-intensified issues, such as the shortages of food and cash, were reported by a majority across states as their livelihoods were disrupted. The impacts were amplified for migrant workers who lacked support systems at the destination and struggled with bureaucratic barriers to accessing local government or non-governmental support.

**Table 5:** Workers’ Issues During Lockdown

Issues (*n* = 272)	Gujarat (*n* = 145)	Uttar Pradesh (*n* = 26)	Maharashtra (*n* = 53)	Kerala (*n* = 48)
Needing rations	132 (91%)	25 (96%)	52 (98%)	38 (79%)
Needing cash	84 (57%)	26 (100%)	36 (67%)	40 (83%)
Charged rent	17 (12%)	–	–	–
Language barriers/Identity-based exclusion	–	18 (69%)	–	45 (93%)
Unaware of helplines	22 (15%)	26 (100%)	13 (25%)	37 (77%)
Unsupported by helplines	48 (33%)	24 (92%)	1 (2%)	–
Faced police hostility	35 (24%)	–	–	–
Supported by employers	–	–	16 (30%)	48 (100%)
Engaged in protest	11 (8%)	–	–	-

Looking at migrant experiences across states, workers interviewed in Maharashtra were stranded along the outskirts of the major cities of Mumbai and Pune. Government and non-governmental organisations offered support to migrant workers within cities, however, they were unable to bear the costs of commuting to the cities. In effect, the only accessible institutions were their employers. While 30% of workers had received money from employers to buy food, the high costs of living meant that money was soon exhausted. One of these workers explained that shops were ‘charging 40 rupees for a kilo of potatoes instead of rupees 20′. Eventually the groups of workers interviewed in both cities found support from workers associations, like truck driver groups, who provided them with some rations.

Workers stranded in Uttar Pradesh also cited not having enough money as a key issue. 69% reported that local workers had received food from government agencies in the past two days, while those from Bihar seemed to be systematically discriminated against. A group of workers stranded in another city needed ‘rations, rice/wheat, or at least cooked food’ however these workers did not own smartphones, had little information on helplines, and didn’t receive any support from them. Both groups were connected by the interviewer to a university organisation that sent them money a few days after their interviews.

Gujarat recorded the highest levels of distress calls. None of the workers had been paid since the lockdown, and employers and contractors had stopped answering their calls. 12% of workers reported being threatened with higher rents by their landlord, in spite of the Ministry of Home Affairs order to the contrary. All the workers interviewed expressed frustration with the implementation of the lockdown which left them helpless and without support. One worker spoke about how at times he felt like crying, while another said the state had left them to die. An NGO staff member of the SWAN group in Gujarat reported their inability to support the increasing number of stranded workers due to lack of resources. By 14th April, many NGOs had been denied permission to function by the state government, and 24% of workers reported being beaten by the police while queuing to access cooked food provided by these organisations. Ironically, workers also reported that they disliked this cooked food, as it was neither nutritious nor filling, but had no money to buy rations.

Kerala was the only state where all workers interviewed received both state and employer support. While 77% of workers were unaware of government helplines, they did not feel the need to use them. Facilities like fuel connections and accommodation were provided by employers. A group of 37 workers who needed rations, got support from the local police station and the panchayat to communicate with the government helpline in the local language, and successfully received rations. All the workers interviewed expressed wanting to return home, but were aware of travel restrictions, hence were prepared to wait.

### Stage 3: Returning Home: the Urgent Need for Social Protection

With all private and public transportation suspended during the lockdown, migrant workers were stranded for close to two months in the cities. In the absence of money or food, evicted from their dwellings, and threatened with violence by both employers and the State, the spectre of desperate migrants walking home became a symbol of class inequality and callousness of the state (Samaddar 2020). On 1st May 2020, the government announced special trains for stranded workers to return home (Thakur 2020), but did not clarify whether fares would be borne by the state or migrant workers (Dhingra 2020). Access was not equitable: workers from most states had to wait for weeks for tickets, most had to pay for them, or had to arrange expensive private vehicles risking penalties as it was in violation of the lockdown rules.

A group of 8 workers trying to return from Uttar Pradesh described the difficulties they faced in accessing transport. They walked 50 km on hearing that the Chief Minister had organized a special bus to Bihar, but due to inaccurate information, reached late and missed the bus. They had to walk back to their rented quarters, but after receiving threats from the police for disobeying the lockdown. On finally boarding a vehicle to Bihar, they explained: ‘It was suffocating, we were 50 passengers crowded in a small truck meant for transporting goods’. Another worker stranded in Gujarat had to wait in large crowds without any water or food to eventually pay 850 rupees, about 25–30% of his normal weekly wage (or 10% of his monthly wage) for a train ticket, at a time, when he was out of work and not receiving wages.

Meanwhile in Maharashtra, a group of 8 workers were unable to secure tickets to return, and finally resumed work in the hope that no further lockdown would be imposed due to the state’s rising COVID-19 cases.

In contrast, the tribal group of workers stranded in Kerala were able to avail of support from state governments in both their destination and Jharkhand state, which shares a border with Bihar. The Collector’s office helped the workers identify the procedures for returning home, enabling them to first get tested for COVID. With negative results, these officials then helped the group register for the Jharkhand government’s special train. ‘The transit was smooth, and the behaviour of the Kerala officials was very humble’, one of the workers said. The train was equipped for the long journey with food and medical facilities.

On returning to their villages, all the workers were aware of quarantine procedures and went to local government quarantine facilities, but found them ill-equipped, with no food, masks or sanitisers. Local authorities blamed the lack of state funding. Villagers and families took over the responsibility for arranging safe accommodation in empty buildings and providing workers with food and other necessities during the 14-day quarantine period.

Meanwhile, on May 13th, announcing the second tranche of the ‘Aatmanirbhar’ (Self-reliance) relief and stimulus package, the Finance Minister, for the first time, identified migrant workers as a distinct group, and announced three measures to address their needs. First, all migrant workers were assured 5 kgs of foodgrains free of cost for two months—May and June—irrespective of their state-based identification. Second, state governments were asked to use funds from allocations to the State Disaster Management Agencies to ensure migrant workers had shelter. In the longer-term, by March 2021, migrant workers have been promised affordable rental housing at their destinations. Finally, more work was being offered to wage-seekers in rural areas under the MGNREGA scheme, at slightly enhanced wage rates, to address the loss of incomes (SNS Web 2020).

While the process of legitimization of migrant workers as a constituency with distinct needs and contestation around the content of these needs unfolded during the lockdown, the third stage reveals a gap between discourse and practice. The relief package recognised migrant workers’ needs for basic survival and livelihood (the right to food, shelter and work), focusing on the levels of provision and prevention (Devereux and Sabates-Wheeler 2008). Yet implementation so far has been poor, as most states distributed less than 2% of the foodgrains allocated to them (Johari 2020). While the demand for work under MGNREGA has almost doubled over the period May–June 2020 compared to previous years (Sengupta 2020), it is unclear how much of the additional demand is from returning migrant workers.

## Discussion

What is clear is that prior to the lockdown, migrant workers and their families considered migration itself a form of social protection. Male migrants additionally gain respect and dignity through their hard work and sacrifice for the family (cf. Rao and Hossain 2014), so as long as they are able to get jobs and earn money, they do not necessarily articulate a demand for state support. In fact, their choice of destination is driven more by their contacts and the availability of work rather than any assessment of norms of fairness and justice. This pride as breadwinners, compounded by the dispersed nature of migrant work, long working hours, lack of ability to associate, and lack of legal awareness, all seem to prevent them from coming together to articulate their needs as a single ‘public’ in Fraser’s terms. As citizens with rights to work and live in any part of the country, their rights are legitimate yet have remained largely invisible and uncontested in social policy.

While social identity matters in shaping migration outcomes (Mosse et al. 2002), in this instance, all migrant workers were similarly vulnerable, deprived of their jobs and incomes, yet unable to return home. For the first time, the social policies of destination states became the key variable shaping their experiences of work, being stranded, and returning home. The lockdown exposed the gaps in social policy, but equally the deeper social cleavages and power relations that hinder access to citizenship rights. While the workers themselves lacked ‘voice’ as a legitimate constituency, their needs claims were presented and contested in the public domain by the media and some civil society actors, what we have called ‘migrant-related publics’. We use Fraser’s (1989) arguments on the politicization, contestation and satisfaction of needs as dependent on the relative voice of different interest groups or ‘publics’, to examine the outcomes in terms of needs satisfaction for the migrant workers, and the extent to which their destination mattered in this process.

The first moment in the ‘politics of needs’ for migrant workers—the legitimisation of needs—took place once the national lockdown was imposed. Migrant workers stranded across destination states were unable to work and as a result were in need of basic social protection. As an unrecognised ‘category’ in destination states, with the exception of Kerala, most workers were left with no mechanisms to access basic needs or seek redressal for non-payment of dues. A month into lockdown, the plight of migrant workers, their social needs and entitlements, while perhaps known, largely remained outside the domain of public policy, with the state still refusing to recognize migrants as a legitimate group with needs to be urgently met. Advocates for migrant workers’ rights called for the formalisation of worker identities through ensuring contracts, regulating working hours and wages, and setting up redressal mechanisms and databases, citing the informality of employment as causing these vulnerabilities (Khandelwal 2020; Pegu 2020).

In the short-term, the absence of state social protection was filled in by various civil society organisations and migrant support networks (Deshingkar et al. 2008). In Maharashtra and Uttar Pradesh, NGOs and workers associations stepped in as the state transferred the responsibility of care to employers, communities and the civil society. In Gujarat, the situation was dire as NGOs were unable to offer support. Whatever little social protection existed, was constructed as charity rather than a right, an affront to their dignity as workers. Desperation here triggered multiple protests by workers, silenced and suppressed by the police (Bhattacharya 2020). Interestingly, migrant voices demanding social protection were loudest in states like Gujarat where the immediate welfare support was weakest. While distress was not necessarily alleviated in the short-run, these protests did set in motion a process of debate and contestation.

The second moment, the contestation of needs, calls into play notions of citizenship, representation and voice. Most migrant workers in India belong to poor and deprived communities, dispersed and with little voice to influence, contest or participate in provisioning in destination states, where they are unregistered, unfamiliar to the city and have limited social networks (Khandelwal 2020). They live and work in hazardous conditions, with poor access to decent housing, healthcare, water and sanitation. At home too, they hesitate to exercise agency and voice to make claims on the state for provisioning of resources to transform their livelihoods (c.f Deneulin 2006). They instead construct their migrant work as a sufficient and dignified way to fulfil promotive, preventive and protective elements of social protection for their families. Yet by the end of the lockdown, with continuous pressure from the media and civil society, migrant workers emerged as a distinct constituency with specific needs and rights.

In terms of the third moment of needs satisfaction, two contradictory trends emerge. The government finally announced a few short-term measures such as boosting rural job schemes or handing out free food to address the migration crisis, but did not assure income security, travel support or compensation for lost work. The announcement of special trains to transport migrant workers, for instance, created an assumption that costs of travel would be borne by the state. Most workers had to however spend their own money, in some cases borrowing for this purpose. It was only from Kerala, that coordination between the two state governments enabled workers to return home free of cost. While these measures address minimum subsistence needs, they reinforce migrant workers as clients of the state and not citizens with rights. With the gradual lifting of the lockdown, there has been a silencing and sidelining of both these short-term needs and the broader claims to social justice.

Moving beyond protective or preventive measures, where a transformative agenda strengthening workers’ rights and ensuring ‘decent work’ conditions could have been initiated, there have been instead a spate of *regressive* legal measures introduced by the State, diluting even the minimum rights and safeguards available to workers. Despite recognition of migrant workers as a legitimate ‘public’ in the discursive arena, the predictions of economic downturn have amplified the voice of other ‘publics’—industrialists and investors. The withholding of adequate service provision to migrant workers by the state alongside a hollowing out of a potentially transformative agenda challenging discrimination and social inequality more broadly could also be interpreted as the voice of powerful economic actors seeking to create and maintain a pool of cheap labour. The interests of migrant workers were sacrificed to capital by the state. As Devereux and McGregor argue (2014, p. 307), the potential of social protection to ‘address social vulnerabilities and contribute to social justice is … not yet widely accepted’. With no options, many of the workers are now returning to their destinations (PTI 2020).

## Conclusion

This paper has sought to answer two questions in relation to the rights of migrant workers in India in the context of the covid-19 pandemic induced lockdown. The first, a more empirical question, focusing on the experiences and encounters of migrant workers across different destination states, has revealed some interesting, though not entirely surprising patterns. While social identity and regional location are seen as established mediators of migrant experiences, in the present context their role and impact was subdued. Social policy frameworks in the destination states, or their absence, played a key role in shaping migrant experience. Kerala, with a better social infrastructure, but also a specific legal and policy framework recognizing the role and contributions of migrant workers as ‘guest workers’, was able to respond more effectively to their needs and demands. Amongst the other states, Maharashtra falls in between: with some recognition of workers’ rights yet divided by a strong movement of regional pride and exclusivity. Gujarat and Uttar Pradesh emerge at the bottom, offering no rights or benefits to the migrant workers, and in fact, denying any claims to citizenship that they put forth.

This analysis raises some issues for the construction of citizenship rights and social policy more broadly. Images of the intensified vulnerabilities confronted by migrant workers across the country during the lockdown have firmly placed them as an emergent ‘public’ with legitimate needs (Fraser 1989). The Constitution of India offers all citizens the freedom to live and work in any part of the country, yet the pandemic exposed the loopholes in ensuring rights in the context of population mobility, placing the spotlight on the nature of rights and their portability. While this is critical, the discursive agenda of social protection is now focused only on basic needs to food and shelter, rather than a transformational agenda that could eventually ensure equal opportunities and choices to all citizens of the country to lead fulfilling lives.

A second key issue relates to the mechanisms for participation and exercise of voice. Migrant workers have largely been silent in the policy space regarding their needs and aspirations—they have sought to fulfil these through the process of migration itself. While the media gave them voice during the lockdown, what became clear nevertheless is their near-total exclusion from local governance systems in the destination states, alongside a near-total blindness by the state to their very existence. Kerala is an exception, as the critical role of local panchayats and decentralized decision-making became evident in alleviating the hardships confronted by the workers post-lockdown. Governance in India, including local governance, is entirely place-based, with migrant workers, despite their significant economic contributions, having no say therein. It is important to establish mechanisms to hear their voices and meet their needs.

A final point in the construction of citizenship rights, and indeed claims to social protection, relates to the gendered construction of need by both the state and society. Men in India pride themselves as ‘providers’, working hard and making sacrifices for the wellbeing of their families. They fear that any claims to social protection may be interpreted as an admission of their inability to successfully perform this role. Women on the contrary are constructed as being more ‘needy’, and hence recognized as the ‘head of households’ for accessing foodgrains as part of their ‘right to food’, and legitimate claimants to unskilled work as part of their ‘right to work’. The problem here appears to be the definition of social protection as a basic need, with women responsible for its fulfilment, rather than its more expansive and universal transformative forms. For this to happen and notions of citizenship enshrined in the Indian Constitution to be realised, social protection measures need to address pre-existing regional and social power inequalities through redistributive measures including strengthening local infrastructure, services (health and education), and participatory governance. Addressing structural inequalities can contribute to expanding choices, strengthening local livelihood opportunities and potentially repositioning the construction of needs in the local context.

Workers may or may not want to migrate to the cities post-pandemic. Rather than regressive measures that seek to exploit their labour, or protective measures that provide for basic needs as ‘charity’, social protection instruments should create a legal framework and social infrastructure that recognises the contributions of migrant workers to the national economy, enables them to envision their own futures and supports them in fulfilling their aspirations. Instead of being viewed as ‘dependent clients’, the right to a life of dignity, as equal and productive citizens, needs to be ensured.
